# Complete Genome Sequencing of *Lactobacillus plantarum* ZLP001, a Potential Probiotic That Enhances Intestinal Epithelial Barrier Function and Defense Against Pathogens in Pigs

**DOI:** 10.3389/fphys.2018.01689

**Published:** 2018-11-27

**Authors:** Wei Zhang, Haifeng Ji, Dongyan Zhang, Hui Liu, Sixin Wang, Jing Wang, Yamin Wang

**Affiliations:** Department of Animal Nutrition, Institute of Animal Husbandry and Veterinary Medicine, Beijing Academy of Agriculture and Forestry Sciences, Beijing, China

**Keywords:** *Lactobacillus planatrum*, complete genome sequencing, intestinal barrier, probiotic, comparative genome analysis

## Introduction

The mammalian gastrointestinal tract is a heterogeneous ecosystem with the most abundant, and one of the most diverse, microbial communities. The gut microbiota, which may contain more than 100 times the number of genes in the human genome, endows the host with beneficial functional features, including colonization resistance, nutrient metabolism, and immune tolerance (Bäckhed, 2005). Dysbiosis of gut microbiota may result in serious adverse consequences for the host, such as neurological disorders, cancer, obesity, malnutrition, inflammatory dysregulation, and susceptibility to pathogens (Turnbaugh et al., 2006; Malo et al., 2010; Wang T. et al., 2012; Subramanian et al., 2014; Sampson et al., 2016).

Lactic acid bacteria (LAB) function as probiotics. *Lactobacillus plantarum*, an important member of the family of LAB, is commonly found in fermented food and as a commensal bacterium in the gut microbiota. The beneficial effects of probiotics are strain-specific (Ramos et al., 2013). Some *L. plantarum* strains were demonstrated to confer various beneficial properties by improving growth performance and promoting gut health, and has been used for the prevention or treatment of various diseases, including inflammatory bowel disease [strain Lp91; (Duary et al., 2012)], uremia [strain AD3; (Patra et al., 2018)] and liver damage [strain C88; (Duan et al., 2018)] in humans and animals. The beneficial effects of *L. plantarum* are associated with the regulation of the immune response [strain 426951; (Soltani et al., 2017)], maintenance of gut microbiota homeostasis [strain ZDY04; (Qiu et al., 2018)], and enhancement of epithelial barrier function [strain 299v; (Barnett et al., 2018)]. In our previous study, *L. plantarum* ZLP001 strain isolated from the gastrointestinal mucosa of a healthy weaned piglet was demonstrated to exhibit high antioxidant ability, and the dietary supplementation of *L. plantarum* ZLP001 was shown to improve growth performance and antioxidant status of weaned piglets (Wang J. et al., 2012). *L. plantarum* ZLP001 inhibits growth and adhesion of enterotoxigenic *Escherichia coli* and enhances host defense by strengthening intestinal epithelial barrier function and innate immune response to secret antimicrobial peptides (Wang J. et al., 2018).

*Lactobacillus plantarum* is a highly flexible and versatile species and has one of the largest genomes among the known LAB. Consistent with the diversity of probiotic functions, complete genome sequencing analysis revealed the genomic diversity and environment specialization of *L. plantarum* strains isolated from different niches, including plant (Liu et al., 2015), dairy (Zhang et al., 2015), or vegetable (Crowley et al., 2013) fermentations, as well as saliva (Kleerebezem et al., 2003) and gut (Li et al., 2013). To gain a better insight into the beneficial effects on gut health in piglets, the complete genome of *L. plantarum* ZLP001 was sequenced and a comparative genome analysis study was conducted between *L. plantarum* ZLP001 and other available *L. plantarum* genomes.

## Materials and methods

### Bacterial growth and DNA extraction

*Lactobacillus plantarum* ZLP001 from single colony was grown in De Man, Rogosa, and Sharpe broth (Oxoid, Hampshire, UK) for 18 h at 37°C under microaerophilic condition. Total genomic DNA was extracted and purified using a Wizard Genomic DNA Purification Kit (Promega, Madison, WI).

### Genome sequencing, assembly, and annotation

A 8–12 kb DNA library was constructed and sequenced using single molecular real-time (SMRT) sequencing technology with C4 chemistry and P6 DNA polymerase on the PacBio RS II system (Pacific Biosciences, Menlo Park, CA) by Shanghai Majorbio Bio-pharm Technology Co., Ltd (Shanghai, China). Raw sequence data were filtered using SMRT Analysis v2.3.0. A total of 73,238 subreads with a median length of 8,661 bp were obtained for *de novo* assembly using the SOAPdenovo v2.04 (Luo et al., 2012). Genes were predicted using Glimmer v3.02 (www.cbcb.umd.edu/software/glimmer) and the corresponding function annotation was completed by blasting genes against Cluster of Orthologous Groups of proteins (COG) and Kyoto Encyclopedia of Genes and Genomes (KEGG) databases. Tandem repeats were predicted using Tandem Repeat Finder v4.04, and the minisatellite and microsatellite DNAs were selected based on the number and length of repeat units. In addition, rRNA, tRNA, and sRNA were predicted using rRNAmmer v1.2, tRNAscan v1.23, and Rfam v10.1, respectively. Genome visualization was performed using Circos v0.69-6. Possible prophage sequences were searched using PHAST (http://phast.wishartlab.com/index.html), and clustered regularly interspaced short palindromic repeats (CRISPR) were predicted using MinCED 3 (https://sourceforge.net/projects/minced/). Carbohydrate active enzymes (CAZymes) were searched against the CAZy database (http://www.cazy.org/).

### Phylogenetic and ortholog clustering analyses

For phylogenetic and comparative genome analyses, a total of 18 complete genome sequences of *L. plantarum* strains were obtained from the NCBI database (Table 1). An orthologous gene set was built to identify the core-genome and pan-genome sizes using OrthoMCL package v2.0 (Li et al., 2003). All predicted protein sequences were merged together and compared with each other using BLASTP algorithm, with an *E*-value cutoff of 1e−5 and a percent match ≥ 50%. All homologous protein pairs were parsed and grouped into orthologous families by cluster tool MCL, with an inflation value of 1.5.

**Table 1:** Genome summary of *Lactobacillus plantarum* strains.

***S*train**	**Source**	**Size (Mb)**	**CDS**	**GC%**	**GenBank No**.	**Probiotic property**	**References**
ZLP001	Weaned piglet gut	3.16	3,264	44.37	CP021086	Inhibit *E. coli* adhesion and maintain tight junction barrier in pigs	Wang J. et al., 2018
WCFS1	Human saliva	3.31	3,042	44.50	AL935263.2	Increase leptin and T cell level to ameliorate high fat diet-induced pathology in mice	Ivanovic et al., 2015
JDM1	Human intestinal tract	3.20	2,948	44.66	CP001617.1	No report
ST-III	Kimchi	3.25	3,013	44.58	CP002222.1	Inhibit growth of *Streptococcus mutans* from children with active caries	Lin et al., 2017
ZJ316	Healthy newborn infant feces	3.20	3,159	44.65	CP004082.1	Improve pig growth and pork quality	Suo et al., 2012
P-8	Fermented raw cow milk	3.03	3,140	44.80	CP005942.2	Improve growth performance and activate intestinal immune response in chickens	Wang et al., 2015
16	Malt production steep water	3.04	2,787	44.74	CP006033.1	Possess potent antifungal activity	Crowley et al., 2012
5-2	Fermented soybean	3.24	3,114	44.70	CP009236.1	Possess high autoaggregation properties and inhibit growth of *Gardnerella vaginalis*	Pessoa et al., 2017
B21	Vietnamese sausage	3.28	2,930	44.47	CP010528.1	Produce bacteriocins against a wide range of Gram-positive bacteria	Golneshin et al., 2015
LZ95	Newborn infant fecal	3.32	2,951	44.49	CP012122.1	Produce extracellular vitamin B12	Li et al., 2017
HFC8	Human gut	3.41	3,447	44.33	CP012650.1	No report
CCUG 36733	Human oral samples	3.05	2,542	44.38	CP014228.1	No report
LZ227	Raw cow milk	3.13	3,262	44.71	CP015857.1	Produce both riboflavin and folate	Li et al., 2016b
LZ206	Raw cow milk	3.21	2,837	44.64	CP015966.1	Possess antimicrobial activity against various pathogens	Li et al., 2016a
KLDS1.0391	Fermented dairy products	2.90	2,902	44.70	CP019348.1	No report
LPL-1	Fermented fish	3.19	2,932	44.65	CP021997.1	Produce class IIa bacteriocin against *Listeria monocytogenes* 54002	Wang Y. et al., 2018
BDGP2	Drosophila melanogaster gut	3.41	3,148	44.24	CP023174.1	No report
10CH	Cheese	3.31	3,192	44.51	CP023728.1	Possess antimicrobial activity against various pathogens	El Halfawy et al., 2017
LQ80	Fermented liquid feed for pigs	3.23	3,186	44.66	CP028977.1	Enhance immune response with increased immunoglobulin A, M and G in pigs	Mizumachi et al., 2009

The phylogenetic tree based on 16S rRNA gene sequences was constructed using the neighbor-joining method by MEGA software. The predicted amino acid sequences of each single copy orthologous gene family were aligned using MAFFT v7 (https://mafft.cbrc.jp/alignment/software/). The individual alignments were concatenated into a string of amino acid sequence alignment and the concatenated alignment data were submitted to RAxML (https://github.com/stamatak/standard-RAxML) to build phylogenomic trees with the maximum-likelihood algorithm. The bootstrap method of 1,000 bootstrap repetitions was used to assess tree reliability.

### Data accession number

The raw and assembled sequence data for *L. plantarum* ZLP001 genome have been deposited at SRA database under the accession number PRJNA381357 (SRP102895) and GenBank under the accession number CP021086, respectively. The strain has been deposited at the China General Microbiological Culture Collection Center (CGMCC no. 7370).

## Results and discussion

### General genome features of *L. plantarum* ZLP001 genome

**Figure 1:**
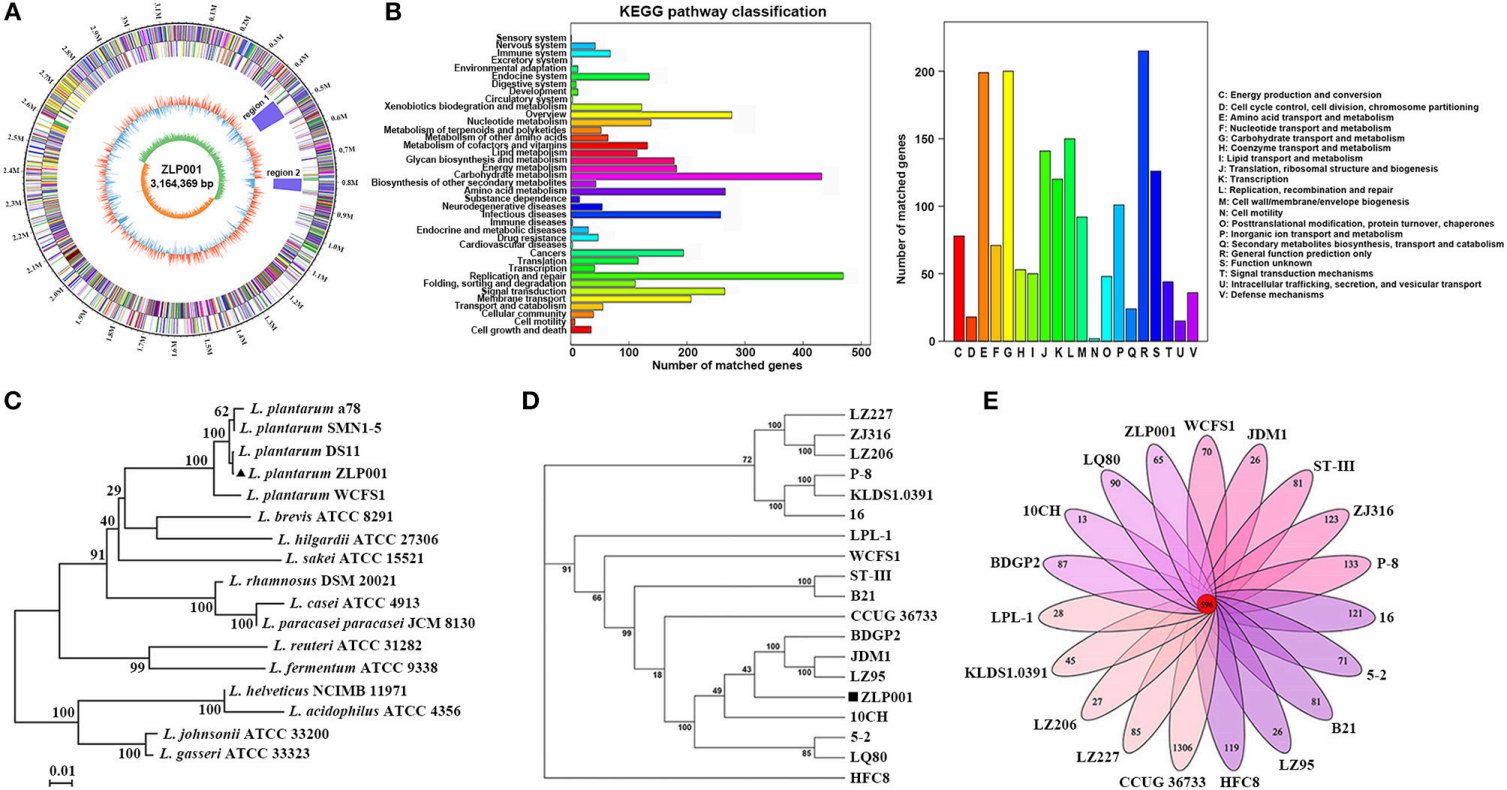
Genome features of *L. plantarum* ZLP001. **(A)** Circular genomic map of *L. plantarum* ZLP001. The circular map was generated using Circos and contains seven circles. Marked information is displayed from the outer circle to the innermost circle as follows: Genome size, CDSs on the forward stand, CDSs on the reverse stand, prophage regions, rRNA and tRNA, GC content, and GC skew. **(B)** Gene number of KEGG and COG categories. **(C)** The neighbor-joining tree of *L. plantarum* ZLP001 based on the 16S rRNA gene sequence. The percent numbers at the nodes indicate the levels of bootstrap support based on neighbor-joining analyses of 1,000 replications. Bar, 0.01 nucleotide substitution per site. **(D)** Phylogenetic tree of 19 *L. plantarum* strains. The phylogenetic tree was built based on aligned concatenated sequences of single copy orthologous gene families. The bootstrap support value before each node represents the confidence degree of each branch. **(E)** Numbers of orthologous gene families and unique genes among 19 *L. plantarum* strains. The Venn diagram shows the number of orthologous gene families of the core genome (the center part) and the numbers of unique genes of each genome. The different colors indicate different sampling areas of the strains as indicated. The orthologous gene families were determined by OrthoMCL software with an inflation value of 1.5.

As shown in Figure 1A, the complete genome of *L. plantarum* ZLP001 contained a single circular chromosome of 3,164,369 bp with a GC content of 44.65% and seven plasmids, namely, A (67,802 bp), B (48,418 bp), C (31,389 bp), D (27,860 bp), E (16,139 bp), F (15,258 bp), and G (13,837 bp), with an average GC content of 42.05%. A total of 3,264 protein-coding sequences (CDSs) were identified. Of these, 3,104 genes with an average length of 886 bp were on the chromosome that occupied 83.28% of the genome and 77, 56, 34, 33, 17, 22, and 21 CDSs with an average length of 614 bp were found in the plasmid ZLP001 plasmid A to plasmid G, respectively. The chromosome contained 16 rRNAs, 69 tRNAs, 91 tandem repeats, 56 minisatellite DNAs, and 7 microsatellite DNAs. A total of 10 sRNAs, 47 tandem repeats, and 27 minisatellite DNAs were found in the plasmids.

### Functional classification

A total of 1,603 CDSs were classified into 39 KEGG functional categories, mainly functioning in replication and repair, carbohydrate metabolism, amino acid metabolism, and signal transduction (Figure 1B). Furthermore, 1,783 CDSs were specifically assigned to clusters of COG comprising 20 categories (Figure 1B). Most genes were classified into function categories for carbohydrate transport and metabolism (200 genes), amino acid transport and metabolism (199 genes), replication, recombination, and repair (150 genes), translation, ribosomal structure, and biogenesis (141 genes), transcription (120 genes), and inorganic ion transport and metabolism (101 genes).

### Phylogenetic relationships among *L. plantarum* strains

As shown in the phylogenetic tree constructed based on the 16S rRNA gene sequence, *L. plantarum* members were grouped and distinguishably separated from other *Lactobacillus* genus strains (Figure 1C). The strain ZLP001 displayed more than 99% similarity with other *L. plantarum* strains based on the 16S rRNA gene sequence. *L. plantarum* genomes were difficult to distinguish by 16S rRNA gene sequence similarity. To further understand the phylogenetic relationship among *L. plantarum* strains, a phylogenetic tree based on the single copy orthologous gene families was built (Figure 1D). A total of 553 single copy orthologous gene families were reported. *L. plantarum* ZLP001 showed a close relationship with the strains BDGP2, JDM1, and LZ95, but was located on a relatively standalone branch. This observation suggests that the strain ZLP001 has a distinguishing pattern of genomic evolution in order to adapt to the gut environment.

### Core- and pan-genomes of *L. plantarum* strains

OrthoMCL results showed that 19 genomes had a pan genome size of 6,598 orthologous gene families and a core genome size of 596 (9.03%) orthologous gene families (Figure 1E and Table S1). A total of 4,141 orthologous gene sets were constructed. The pan genome size was 1.36 times the average size of these 19 genomes and the core genome constituted 19.59% of each *L. plantarum* genome. A total of 2,597 (39.36%) strain-specific genes were found, and the number of strain-specific genes ranged from 13 genes only in strain 10CH to 1,306 genes unique to strain CCUG36733. A total of 65 genes were unique to ZLP001 strain (Table S2), including 30 genes encoding hypothetical proteins. The formation of a large gene pool for these *L. plantarum* members implies their open pan genome structures. This property, to a certain extent, endows these members with the capability of adaptation to the surrounding environments or their hosts.

### Lifestyle adaptation to stress

*Lactobacillus plantarum* ZLP001 carries several genes encoding stress-related proteins. ZLP001 encoded genes for Na^+^:H^+^ antiporter and choloylglycine hydrolase, providing the evidences of the tolerance of *L. plantarum* ZLP001 to low pH and bile salt in the gastrointestinal environment. *L. plantarum* ZLP001 also carried various genes encoding heat stress proteins, including heat shock protein 10 (Hsp20, gene 0190, gene 1178, gene 2786, and gene 2876) and Hsp33 (gene 0295, gene 0414, gene 1058, and gene 2105), heat shock protein Htpx (gene 0076, gene 0632, gene 1622, gene 1079, and gene 2136), and molecular chaperone DnaK (gene 0299, gene 0873, gene 0901, gene 1193, and gene 1501). The whole genome analysis suggests the strong potential of *L. plantarum* ZLP001 proliferation and genetic tolerance and lifestyle adaptation to detrimental stress in the gut.

### Transport and secretion system

*Lactobacillus plantarum* ZLP001 genome harbored 306 genes related to transport system, which mainly constitutes the phosphotransferase system (PTS) and ATP-binding cassette (ABC) transporter system (Table S3).

Of these transporters, 54 genes were related to the genomic PTS system. *ptsH* (gene 1521) encoded for the phosphocarrier protein HPr, which delivered phosphoryl groups from phosphoenol pyruvate to PTS EII enzymes (EIIs). A total of 10 complete phosphoenolpyruvate-dependent PTS EII complexes were present in ZLP001 genome that were involved in the transport of carbon sources, including β-glucosides, cellobiose, fructose, galactitol, glucose, mannitol, mannose, N-acetylgalactosamine, sorbitol, and sucrose. These sugar PTS systems have the ability to import more than one substrate and expand the carbon transport capacity of *L. plantarum*.

A total of 252 genes involved in the genomic ABC transporter system components were found in ZLP001 genome. Many of these ABC importers transport inorganic ions, peptides, and amino acids, whereas the substrate specificity of most of the exporters was unknown, as described for *L. plantarum* WCSF1 (Kleerebezem et al., 2003) and 5–2 (Liu et al., 2015). The ZLP001 chromosome harbored 10 transporters for the uptake of branched-chain amino acids, including an ABC transporter encoded by the *livHKM* genes (gene 3099 to gene 3102), which were more than those reported in 5-2 genome (5 transporters). Several major facilitator superfamily transporters were also found in ZLP001 genome. Two complete glutamine-specific systems (gene 0770 to gene 0771 and gene 1837 to gene 1840) were found in ZLP001 genome, which are lesser than those reported in the genomes of *L. plantarum* WCSF1 and 5-2. However, one gene (gene 0854) encoding nitrate ABC transporter was unique in the ZLP001 genome. Among LAB, nitrite reduction was demonstrated to occur in *L. plantarum* (Paik and Lee, 2014). *L. plantarum* ZLP001 may play an important role in the regulation of nitrogen metabolism via glutamine synthetase and nitrite reduction.

The Sec-SRP secretion system was found on the chromosome of *L. plantarum* ZLP001 genome, including the signal-recognition particle proteins Ffh (gene 1224), membrane protein YidC (gene 1301 and gene 2562), and the component SecA (gene 1968), SecE (gene 2015 and gene 2026), SecG (gene 1917), SecY (gene 1672), and YajC (gene 0596). In addition, the type IV secretion system was present in the plasmids of *L. plantarum* ZLP001, comprising three VirD4 proteins in plasmid F (encoded by gene 0001 and gene 0019) and plasmid G (encoded by gene 0015).

### Mobile genetic element analysis

The ZLP001 genome contained two intact prophage elements (Figure 1A and Table S4). One prophage region resembled Lactob_Lj965 (82.1 kb, region 1) with a GC content of 42.27%, while the other resembled Lactoc_lato (52.7 kb, region 2) with a GC content of 40.18%. The closest related phage for *L. plantarum* was phig1e (Desiere et al., 2002). Integrases are useful markers for prophages in bacterial genomes. Three integrases (genes 0417, 0040, and 0801) were identified in the prophage region 1 and region 2. Prophage region 1 extended from 437,762 to 519,876 bp and contained 105 CDSs, with a complete prophage element from gene 0417 (phage integrase) to gene 0521. Prophage region 2 extended from 786,842 to 839,563 bp and carried 60 CDSs, with a complete prophage element from gene 0801 (phage integrase) to gene 0860. Consistent with the findings reported for *L. plantarum* WCSF1 and 5–2, the two intact prophage elements carried the entire packaging/head/tail gene clusters and lysis cassette (14), DNA packaging genes (encoding small and large terminase, portal protein), and head genes (encoding major head protein), as well as tail genes (Liu et al., 2015).

*Lactobacillus plantarum* ZLP001 genome contained 22 CRISPR loci (CRISPR 1 to CRISPR 22), including 9 CRISPR loci in the chromosome and 13 CRISPR loci in the plasmids (Table S5). The detected CRISPR/CRISPR-associated (Cas) system in plasmid C was type II-A (four cas genes; cas 1, cas 2, cas 9, and csn 2), consistent with the previously described CRISPR loci characteristic of *L. rhamnosus* Pen (Jarocki et al., 2018).

### CAZymes

The analysis of CAZymes showed that the ZLP001 genome contained 119 genes in the five CAZymes gene families (Table S6) as follows: 18 carbohydrate esterase (CE) genes, 13 carbohydrate-binding modules (CBMs), 32 glycosyl transferase (GT) genes, 50 glycoside hydrolase (GH) genes, and 6 auxiliary activity (AA) genes. These numbers of CAZymes were relatively smaller than those for *L. plantarum* KLDS1.0391. *L. plantarum* KLDS1.0391 harbors only 14 CEs, 21 CPMs, 23 GTs, 34 GHs, and 2 AAs (Jia et al., 2017). GTs may catalyze the transfer of sugars from the activated donor molecules to specific acceptors and are essential for the formation of surface structures recognized by the host immune system (Mazmanian et al., 2008). The higher number of genes encoding CAZymes in *L. plantarum* ZLP001 genome is suggestive of its probiotic potential for pathogen defense and immune stimulation.

### Genes related to antioxidative capacity

Excessive accumulation of reactive oxygen species (ROS) and reactive nitrogen species (RNS) during cellular metabolism results in oxidative stress and leads to oxidative damage to proteins, lipids, and nucleic acids. *L. plantarum* ZLP001 encoded genes for some proteases involved in stress response, such as the ATP-dependent intracellular proteases ClpP (gene1927) and HslV (gene1042), which prevent aberrant damage to proteins (Table S7). LAB species with a complete glutathione (GSH) system may directly detoxify hydrogen peroxide and lipid peroxyl radicals through the regulation of the protein dithiol/disulfide balance (Pophaly et al., 2012). We identified a series of genes for GSH redox reaction, including *gpx* coding GSH peroxidase (gene 2371) and *gor* coding GSH reductase (genes 1060, 1539, 2244, and 2804). GSH-dependent disulfide reductions are also catalyzed by the thioredoxin system and glutaredoxin. The thioredoxin system provides electrons to thiol-dependent peroxidases to remove ROS and RNS at high reaction rates (Lu and Holmgren, 2014). *L. plantarum* ZLP001 harbored the genes related to the complete thioredoxin system, including five *trxA* genes coding thioredoxin (genes 0579, 0606, 2352, 2358, and 2727), one *trxB* gene encoding thioredoxin reductase (gene 1948), and one *tpx* gene coding thiol peroxidase (gene 0564). The gene *nrdH* encoding glutaredoxin (gene 2011) was also identified.

Bacteria regulate the levels of ROS and RNS with enzymatic and non-enzymatic cellular defense mechanisms. The gene *kat* (gene 2622) encoding catalase was shown to catalyze the decomposition of hydrogen peroxide to non-toxic water. *L. plantarum* ZLP001 genome encoded various nicotinamide adenine dinucleotide (NADH) oxidation-related proteins, including three *nox2* genes encoding NADH oxidase (genes 0956, 1949, and 2717) and two *npr* genes encoding NADH peroxidase (genes 0354 and 1370). Catalase and NADH oxidase/peroxidase are directly implicated in hydrogen peroxide and ROS degradation. Consistent with the observations in other *L. plantarum* strains (Kleerebezem et al., 2003; Liu et al., 2015), *L. plantarum* ZLP001 had no genes encoding the enzyme superoxide dismutase. In *L. plantarum*, nonheme type II catalase contains a dinuclear manganese active site. Crystallography analysis showed that the manganese active sites include μ_1, 3_-bridging glutamate carboxylate residues that appears to be unique to *L. plantarum* and are involved in the transfer of the peroxidic protons to active site bases (Barynin et al., 2001). We found that *L. plantarum* ZLP001 carried the gene *aspB* (genes 0071, 1136, and 2904) encoding aspartate aminotransferase, while the same gene was absent from other sequenced *L. plantarum* strains such as *L. plantarum* strain WCFS1 (Kleerebezem et al., 2003), JDM1 (Zhang et al., 2009), and ZJ316 (Li et al., 2013). Aspartate aminotransferase catalyzes the production of glutamate from oxoglutarate, which could enhance the activity of manganese active site in catalase and strengthen *L. plantarum* ZLP001 tolerance to oxidative stress.

*Lactobacillus plantarum* ZLP001 is a potential probiotic with antioxidative capacity and is known to enhance the intestinal epithelial barrier function and defense against pathogens. The present study provides a genomic overview and reports the distinguishing gene features of ZLP001 by comparative genomic analysis of 18 related strains. The primary finding of this study requires further confirmation through *in vivo* and *in vitro* studies. The complete genome sequence of *L. plantarum* ZLP001 may improve our understanding of the probiotic effects of *L. plantarum* ZLP001 and extend its potential applications in humans and animals.

## Author contributions

WZ and HJ conceived and designed the experiments. SW, JW, and YW performed the ZLP001 cultivation, and DNA extraction. WZ, DZ, and HL performed the genome analysis. WZ and HJ prepared the manuscript.

### Conflict of interest statement

The authors declare that the research was conducted in the absence of any commercial or financial relationships that could be construed as a potential conflict of interest.
