# Supplementary material for: Complete Genome Sequencing of Lactobacillus plantarum ZLP001, a Potential Probiotic That Enhances Intestinal Epithelial Barrier Function and Defense Against Pathogens in Pigs
Source: Front Physiol. 2018 Nov 27;9:1689. doi: 10.3389/fphys.2018.01689 (PMC6277807; doi:10.3389/fphys.2018.01689)
Supplement: Supplementary file 7 [file Table_7.DOCX]

**Table S7 Comparison of genes related to antioxidative capacity in *L. plantarum* ZLP001 and other members of *L. plantarum*.**

| **Gene** | **Predicted function^a^** | **ZLP001^b^** | **WCFS1** | **ZJ316** |
| --- | --- | --- | --- | --- |
| *ClpP* | intracellular proteases | O | O | O |
| *HslV* | intracellular proteases | O | O | O |
| *kat* | catalase | O | O | O |
| *nox2* | NADH oxidase | O | O | O |
| *npr* | NADH peroxidase | O | O | O |
| *aspB* | aspartate aminotransferase | O | X | X |
| *gpo* | GSH peroxidase | O | O | O |
| *gsr* | GSH reductase | O | O | O |
| *trxA* | thioredoxin | O | O | O |
| *trxB* | thioredoxin reductase | O | O | O |
| *tpx* | thiol peroxidase | O | O | O |
| *nrdH* | glutaredoxin | O | O | O |

^a^ NADH: nicotinamide-adenine dinucleotide, GSH: glutathione.

^b^ O: detected, X: not detected.
